# The impact of agricultural disasters on child development in rural China

**DOI:** 10.3389/fpubh.2022.952734

**Published:** 2022-11-02

**Authors:** Sipei Xu, Peng Zhan

**Affiliations:** ^1^School of Public Affairs, Zhejiang University, Hangzhou, China; ^2^Institute for Common Prosperity and Development, Zhejiang University, Hangzhou, China

**Keywords:** agriculture disasters, rural children's development, cognitive skills, non-cognitive skills, academic pressure

## Abstract

**Background:**

China's uneven development under the urban-rural dichotomy has led to the discouraging development of children in rural areas. China is a large agricultural country and agricultural disasters are relatively common. Rural children aged 10–15 whose families depend on the agricultural economy may experience far-reaching negative effects from these disasters.

**Objective:**

This study explored the effects of agricultural disasters on rural children's development, including cognitive and noncognitive skills, and academic pressure.

**Methods:**

Survey data from the China Family Panel Survey and the National Meteorological Administration for 2010–2018 and a fixed-effect panel model with difference-in-differences regressions were used in the study.

**Results:**

The fixed effects model results showed evidence that agricultural disasters have a negative impact on rural children's cognitive and noncognitive skills and a positive impact on academic pressure. The statistically significant coefficients are −0.092, −0.938, and 0.223, respectively. School and family environments also explain children's development. Robustness tests confirmed these results.

**Conclusions:**

Evidence shows that agricultural disasters have a significant negative impact on rural child development. It may be inferred that these will increase the difficulty of narrowing the urban-rural development gap. China is committed to promoting prosperity for all its people. Special attention should be paid to the consequences of disasters at the child level and appropriate measures should be taken to mitigate possible negative impacts.

## Introduction

Agricultural disasters have a significant impact on farming families because of their uncertain and destructive nature (1). With global climate change, their more frequent occurrence poses a great threat to the sustainable development, life, and well-being of rural families (2), a threat that has drawn global attention (3–5). China is an important agricultural trading country in the world, and has a long agricultural production cycle, unstable economic productivity, and high risks in production. It suffers the most serious agricultural natural disasters in the world, with many types of disasters, large affected areas, and a high proportion of disasters over the years (6). Agricultural production in China is highly affected by drought (7), floods (8), spring forests (9), insect pests (10), and meteorological disasters (11). Many studies have demonstrated the direct impact of natural disasters on farming, such as physical damage to crops and livestock production, leading to harvest failures and reduced revenue from animal husbandry (12, 13). However, information on the indirect impacts of agricultural disasters, especially on children in rural China, is limited; however, several studies have noted the adverse effects of agricultural volatility on the livelihoods of rural families (14), which in turn have serious effects on critical investments in rural children such as school enrollment and malnutrition (15). These findings need further verification. Moreover, the effect of agricultural disasters on other manifestations of child development, such as cognitive and noncognitive skills, has largely been ignored, another area for further investigation.

We think that the likely impact of agricultural disasters on child development is primarily a further consequence of disasters' shocks to household income and consumption. Taylor (16) found that the choice of consumption behavior is quite different with the variation in the income loss. Later studies have further proved that when faced with the impact of natural disasters, the consumption smoothing and consumption insurance will lead families to make different consumption choices when faced with disasters. Harbaugh (17) shows that people who have suffered from severe famine will form a long-term frugal consumption habit, and the consumption level will increase slowly. Moreover, the experience of serious disasters will lead to the formation of high saving tendency, which will lead to insufficient motivation of private investment and consumption. Chetty (18) used the Indonesia and the United States data and found that external shocks, such as disasters, have great impact on household consumption decisions. Families with poor economic conditions are more likely to maintain the basic consumption level by cutting children's human capital investment.

Child development is a lasting subject that has received worldwide attention and importance for up to a century (19, 20); however, uneven child development continues to be widespread worldwide, especially in developing countries (21–23). As the world's largest developing country, China's uneven development across the urban-rural dichotomy has led to disappointing development of children in rural areas (24, 25). Research has shown that the uneven development of children in rural China needs to be taken seriously (26), especially regarding their academic performance (27) and cognitive and noncognitive skills (28). Children's academic performance impacts their future educational attainment and health (29), associated with an increase in income and social status in the long-term (30). An increasing number of studies have explored the academic performance of children as an important way to promote the development of human capital (31–33), which may not only promote children's self-development (34) but also improve the subjective well-being of their parents (35). However, the academic performance of rural children is very different from that of urban children and is closely related to family income (36). In rural China, because of limited family resources (37), parents of poor families are usually unable to sufficiently invest in their children's education (38), which then affects their children's achievements. Studies have also found the differences between rural and urban children when it comes to cognitive and noncognitive skills are significant (39, 40). Since cognitive and noncognitive skills are regarded as strong determinants of employment status, work experience, and wages, they are important predictors of schooling outcomes and have been shown to strongly correlate with engagement in a range of risky behaviors (such as smoking, teenage pregnancy, and crime) (41). Consequently, the improvement of rural children's cognitive and noncognitive skills also needs to be considered. Studies have shown that many factors influence children's outcomes, such as parental education (42, 43), parental effort (44–46), school and teacher quality (47–49), and socioeconomic background (50–52), etc. However, studies of external environmental condition factors, such as agricultural disasters, which occur frequently in rural areas and have a significant impact on the lives of rural families in the Chinese context, remain limited.

This study aimed to explore the effect of agricultural disasters on rural children's outcomes (academic performance, cognitive, and noncognitive skills) and to contribute to the existing literature in the following ways. First, as existing studies have mainly focused on the direct impact of agricultural disasters on agricultural development and farm household income, we further explore the indirect impact of agricultural disasters on rural children's development from the perspective of academic performance and cognitive and noncognitive skills. Second, research on children's development has not focused on agricultural disasters, although some studies found that earthquakes have psychological repercussions and noncognitive impairment in children (53), and school closures due to forest fires affect children's academic performance (54); studies have only focused on natural disasters in other countries, but not on agricultural disasters in China. This study focuses on the impact of agricultural disasters on rural children and provides a new perspective for agricultural policy research. Third, this study analyzed longitudinal data on rural children in China, and hence this is one the first studies to evaluate and examine the effects of agricultural disasters on children's development using panel data measuring current and deferred impacts. Finally, this study used difference-in-differences regression to assess the relationship between agricultural disasters and rural children's development to mitigate endogeneity problems caused by selection bias.

Given that households in rural settings whose income mainly comes from agriculture often face significant risks of agricultural disasters, and most rural children's development will be influenced by the daily production and operation of their households. Since this is relevant to future human capital development and productivity gains, one might expect study of the absolute and relative importance of children's noncognitive skills to be greater in developing countries. However, high cognitive skills and academic performance may be relatively scarce in such settings. Thus, empirical evidence of the absolute and relative importance of children's cognitive and noncognitive skills and academic performance in developing countries is of considerable interest.

## Materials and methods

### Data

The data came from China Family Panel Studies (CFPS), which focuses on the economic and non-economic welfare of Chinese residents and many research topics, including economic activities, educational achievements, family relations and family dynamics, population migration, and health. This is a nationwide, large-scale, multidisciplinary social tracking survey project. The sample covered 25 provinces and autonomous regions, with a target sample size of 16,000 households. The children questionnaires, adult questionnaires, and the family economy questionnaires were available in all surveys of 2010, 2012, 2014, 2016, and 2018. The five waves of cross-sectional data were then combined into imbalanced panel data. We retained variables such as cognitive ability, noncognitive ability, academic pressure, children's age, children's gender, whether there is mountainous terrain in the locality, father's age, mother's age, father's education level, mother's education level, and deleted the missing samples.

The agricultural disaster data came from agricultural disaster reports published by the National Meteorological Administration, including variables such as the year of natural disaster occurrence, provinces, regions, cities, and counties, disaster types, affected crops, date of occurrence, and degree of disaster. We merged household and agricultural disaster data by year and county. The final sample size was 5,188, including 1,593 children in 2010, 862 in 2012, 1,075 in 2014, 882 in 2016, and 776 in 2018.

### Model setting

Owing to the repeated occurrence of agricultural disasters, natural experiments with multiple occurrences are suitable subjects for analysis. Several studies have been conducted to assess the impact of natural disasters using the difference-in-difference method (55–57). This study refers to Raker's work (58) and uses the difference-in-differences method for multi-period quasi-natural experiments to separate the impact of agricultural disasters on the development of rural children from the time series of influencing factors to assess the impact of agricultural disasters on the development of rural children. Compared with the occurrence of a single event, the temporally staggered occurrence of agricultural disasters in this study requires a certain degree of exclusion of other potentially confounding variables from interfering with the results. To clearly capture the impact of the occurrence of agriculture disasters on the development of rural children, children in counties where agriculture disasters occurred between 2010 and 2018 were used as the treatment group and where agriculture disasters did not occur, those children were used as the control group, thus enabling the impact of the quasi-natural experimental event of “agriculture disasters” on the development of rural children to be obtained. We used unbalanced panel data for our empirical analysis. The fixed effect panel model equation is shown in Equation (1).


(1) $$\begin{array}{l} Y_{it}=\alpha_{i}+\beta_{1}Disaster_{i,t}+\delta_{t}+\varphi_{i}+\gamma Z_{i,t}+\varepsilon_{i,t} \end{array}$$

In Equation (1), the dependent variables *Y*_*it*_ are cognitive skills, noncognitive skills, academic pressure, and academic achievement. The core independent variable *Disaster*_*i, t*_ is equal to 1 if the child's county has been hit by any agricultural disaster in the period from 2010 to year t and 0 if no agricultural disaster occurred during that period. For example, if the children's county was hit by an agricultural disaster in 2014, the values of *Disaster*_*i, t*_ are 1 in 2014, 2016, and 2018 and 0 in 2012. Since the difference-in-difference method involves constructing a “treatment group” with a policy treatment and a “control group” without a policy treatment, and it can explain the policy effects by controlling for other factors and comparing the differences between the treatment and control groups before and after the implementation of the policy, it is widely used to evaluate policy effects (55, 56). Moreover, as in our study, there is a sequential difference in the timing of the “treatment” of the study subjects, with the policy starting as a pilot and gradually being rolled out, which constitutes a progressive difference-in-difference model. Other uses of this model include using the incremental push for democratization in different African countries to examine its impact on infant mortality (57), using the progressive diffusion of the family land contract responsibility system there, to examine its impact on the progressive diffusion of the family land contract responsibility system in different countries (55); another use is to track bank deregulation in different North American states to examine its impact on income distribution (58).

*Z*_*i, t*_ are the control variables, including children's birth year, gender, the presence of high mountainous terrain, and the timing of fights with parents. δ_*t*_ indexes the time fixed effects, and φ_*i*_ indexes the area fixed effects.

### Agricultural disasters in China

Since China is one of the countries most affected by agricultural disasters in the world, such disasters have severely affected the production and livelihoods in rural areas during 2009–2018 (59). According to Agri-*meteorological* disasters, hazards, drought, wind and hail, and low-temperature frost damage rates all show a decreasing trend, whereas floods do not show a clear upward trend (60, 61). The most obvious characteristics of agricultural disasters in China are their prevalence, regional nature, and seasonality (62). Droughts are significantly greater in the north than in the south; the west and north-west are vulnerable to wind and hail disasters, and the Yangtze River Basin and the three northeastern provinces are more affected by floods, which are universal, regional, destructive, and defendable (61, 62). Low-temperature frost is more severe in the north than in the south, and wind and hail damage is likely to have sustained and severe impacts in the northwest (61). According to Agri-*biological* disasters, more than 1,700 species that occur year-round and more than 100 periodically prevalent can cause serious damage (63). Agri-biological disasters vary between provinces. Ten provinces have been more affected by crop pests and diseases than the national average, with the top four provinces being Shanghai, Jiangsu, Zhejiang, and Hebei, in order of severity. There were 16 provinces in which weed damage to farmland was heavier than the national average: the six provinces with the most severe weed infestations were Shandong, Hunan, Jiangsu, Hebei, Beijing, and Zhejiang. Rodent infestation on farmland was heavier than the national average in 15 provinces, of which four provinces were heavily affected, including Beijing, Jilin, Qinghai, and Chongqing (64).

The occurrence of agricultural disasters was used as an independent variable in this study. According to the statistics on agricultural disasters published on the official website of the National Meteorological Administration, during 2009–2018, 48 county districts in 39 municipalities, including 19 provinces directly under the central government, suffered agricultural disasters and losses. Agricultural disasters include agri-meteorological and agri-biological disasters, such as rainstorms, continuous rain, hail, drought, snow damage, cold damage, freezing damage, frost, cold wind, strong winds, dry-hot winds, high temperature and high-temperature heat damage, floods, wind disasters, tropical cyclones, stripe rust, powdery mildew, sheath blight, scabs, rice blast, borer, and rice planthoppers. The affected crops include grain crops, such as early rice, late rice, and spring wheat, as well as cash crops, such as sunflower, peanut, and ordinary cotton. The degrees of agricultural disasters were light, medium, heavy, and unknown. The occurrence of agricultural disasters is an independent variable, and has a balanced and gentle trend over time. Agricultural disasters affected 220 children in 2010, 128 in 2012, 126 in 2014, 126 in 2016, and 97 in 2018.

### Dependent variables

The dependent variables were rural children's cognitive skills, noncognitive skills, and academic pressure.

(1) Cognitive skills were recoded as 1 for good and 0 for bad. The measurement of cognitive skills involved standardizing the scores of the math test and word test in the cognitive ability part of the CFPS questionnaire and then adding them together to obtain the total cognitive ability score. The top 50% of cognitive skills was recorded as 1, and the bottom 50% were recorded as 0.
(2) Noncognitive skills were based on a scale of 10, which corresponded to 0–10 as low to high. Noncognitive skills were measured by summing up the scores of the items of the CES_D scale in the noncognitive ability part of the CFPS questionnaire. The CES_D scale is called Catchment-area Epidemiology Survey-Depression Scale, which is widely used in epidemiological investigation to screen out people with depressive symptoms. The higher the scale score reached, the worse the mental health was.
(3) Academic pressure reflects the academic performance of rural children. It was measured using a five-level question in the academic performance section of the CFPS: “What level do you think your academic pressure is?” Options 1 and 2 were coded as 0, and options 3, 4, and 5 were coded as 1.

The three dependent variables were cognitive skills, noncognitive skills, and academic pressure. Descriptive statistics showed that rural children's development differed depending on whether their district was experiencing agricultural disasters. To further verify whether agricultural disasters have an impact on rural children, the following results were obtained.

## Results

### Basic results

Table 1 shows the impact of agricultural disasters on rural children's development using a fixed-effect panel model with difference-in-difference regression. For comparison, we used ordinary least squares (OLS) regression at the same time, which indicated that agricultural disasters had no statistical correlation with rural children's cognitive and noncognitive skills or academic pressure. However, there was a possible causality in the difference-in-differences models controlling for individual fixed effects.

**Table 1:** DID regressions results.

**Variable**	**Cognitive** **skill**	**Noncognitive** **skill**	**Academic** **pressure**
agriculture disasters occurring	−0.092^*^	−0.938^***^	0.223^*^
	(−2.39)	(−5.66)	(2.12)
children gender^*^time	−0.009	0.070	−0.051^*^
	(−0.46)	(1.18)	(−2.12)
childbirth^*^time	0.025^***^	0.091^***^	−0.005
	(5.34)	(5.01)	(−0.90)
dadbirth^*^time	0.001	0.018	0.003
	(0.06)	(1.95)	(0.87)
dad education year^*^time	−0.002	−0.021^**^	0.003
	(−0.48)	(−2.62)	(1.04)
mombirth^*^time	0.004	−0.023^*^	−0.001
	(1.22)	(−2.03)	(−0.03)
mom education year^*^time	−0.006^*^	0.010	0.001
	(−2.37)	(1.29)	(0.06)
mountainous terrain^*^time	0.006	−0.130	−0.024
	(0.27)	(−1.78)	(−0.78)
_cons	0.173^***^	6.594^***^	0.495^***^
	(7.78)	(80.32)	(17.66)
Obs	5,188	5,188	5,188
Adj R-square	0.272	0.120	0.067

* *p* < 0.1,

** *p* < 0.05,

*** *p* < 0.001.

**First**, agricultural disasters negatively impact rural children's cognitive skills. When the district of children's families has been affected by agricultural disasters in the last 2 years, children's cognitive scores (including math skills and word skills) will have a distinctive decrease. The coefficient of agricultural disasters occurring is 0.092 and significantly negative at the 10% level when it comes to rural children's cognitive skills, which indicates that the occurrence of agricultural disasters significantly reduces the cognitive skill of rural children, in terms of economic significance; if the municipality that children live in suffered agricultural disasters in the last 2 years, the scores of their cognitive skills would decrease by 0.092 points.

**Second**, agricultural disasters negatively affected rural children's noncognitive skills. When the district of children's families has been affected by agricultural disasters in the last 2 years, the children's noncognitive scores (CES_D scores) decrease. The coefficient of agricultural disasters occurring is 0.938 and significantly negative at the 0.1% level, which indicates that if the municipality in which children live suffered agricultural disasters in the last 2 years, the scores of their non-cognitive skills decreased by 0.938 points.

**Third**, agricultural disasters negatively impact rural children's academic pressure. When a district of children's families has been affected by agricultural disasters in the last 2 years, the children's academic pressure will increase. The coefficient of agricultural disasters occurring is 0.223 and significantly positive at the 10% level, which illustrates that if the municipality that children live in suffered agricultural disasters in the last 2 years, the level of their academic pressure would increase by 0.223 points, that is, the occurrence of natural disasters significantly increases the academic pressure on rural children.

The results of the estimation of the other control variables were also consistent with theoretical expectations. In summary, the occurrence of agricultural disasters had a significant negative impact on rural children's cognitive and noncognitive skills and a positive impact on academic stress.

As for the OLS regression results, there were no significant results on the impact of agricultural disasters on rural children's development, but all had the same direction of coefficients as the fixed affected panel model results. These results indicate that, although there were no statistical relationships between agricultural disasters and rural children's cognitive and noncognitive skills, the fixed affected panel model results proved that causality existed between them.

### Robustness test

To test the robustness of the results and further reduce endogeneity problems, we firstly tested parallel trend assumptions as the approach used in similar literature. They are consistent with the above results. Then, we selected children who appeared three times in the full sample, giving us five cross-sectional samples to form a subsample. The sample size was 1125. The fixed affected panel model with difference-in-differences regression, as seen in equation (1), was also used to estimate the impact of agricultural disasters on rural children's development. Table 2 shows the results of the three fixed affected panel models with children's cognitive skills, noncognitive skills, and academic pressure.

**Table 2:** DID regression result of subsample regression of appearances 3 times.

**Variable**	**Cognitive** **skill**	**Noncognitive** **skill**	**Academic** **pressure**
agriculture disasters occurring	−0.102^*^	−1.189^***^	0.205
	(−2.21)	(−6.55)	(1.14)
children gender^*^time	−0.015	−0.022	−0.053
	(−0.63)	(−0.27)	(−1.43)
childbirth^*^time	0.029^***^	−0.068^*^	0.008
	(3.73)	(−2.36)	(0.75)
dadbirth^*^time	−0.001	0.027	0.001
	(−0.16)	(1.62)	(0.15)
dad education year^*^time	0.001	−0.003	0.002
	(0.31)	(−0.34)	(0.53)
mombirth^*^time	0.007	−0.023	0.005
	(1.55)	(−1.34)	(0.78)
mom education year^*^time	−0.005	−0.004	−0.001
	(−1.27)	(−0.43)	(−0.15)
mountainous terrain^*^time	−0.045	−0.121	−0.015
	(−1.76)	(−1.36)	(−0.37)
_cons	0.198^***^	6.698^***^	0.433^***^
	(8.31)	(73.98)	(13.45)
Obs	1,125	1,125	1,125
Adj R-square	0.328	0.107	0.089

* *p* < 0.1,

**p < 0.05,

*** *p* < 0.001.

According to children's cognitive and noncognitive skills, the results in Table 2 are similar to those in Table 1. First, for children's cognitive skill level, the coefficient of agricultural disasters occurring is −0.102 significantly negative at the 10% statistical level, that is, when the municipality in which children live in suffered agriculture disasters in the last 2 years, the scores of their cognitive skill would decrease by 0.102 points, which indicates that the occurrence of natural disasters significantly reduces the cognitive skill of rural children; second, for children's noncognitive skill level, the coefficient of agricultural disasters occurring is −1.189 significantly negative at the 0.1% statistical level, indicating that the occurrence of natural disasters significantly reduces the noncognitive skill of rural children. These robustness test results again confirm the cognitive and noncognitive skills results of the full-sample regression.

### Heterogeneity

#### School class types

As children's development may be influenced by their school and class environments, this study analyzed the heterogeneity of children from different class types affected by agricultural disasters. Some schools assign students to key or non-key classes. These schools usually place more emphasis on students' academic performance, and students in key classes tend to have a more solid foundation, generally better grades and better teachers, but also more pressure; in contrast, students in non-key classes have poorer grades and a poorer learning atmosphere. Some schools do not differentiate between key and non-key classes, and all students are in ordinary classes. According to the differences in class types, children were divided into three groups: key, non-key, and ordinary classes. The results of class-type heterogeneity analyses are presented in Table 3.

**Table 3:** The results of class types heterogeneity analysis.

**Class types**	**Key class**			**Non-key class**			**Ordinary class**		
**Variable**	**Cognitive** **skill**	**Noncognitive** **skill**	**Academic** **pressure**	**Cognitive** **skill**	**Noncognitive** **skill**	**Academic** **pressure**	**Cognitive** **skill**	**Noncognitive** **skill**	**Academic** **pressure**
agriculture disasters occurring	0.531	−2.141	0.300	−0.131	−2.153^***^	0.782^***^	−0.028	−0.630	0.121
	(1.72)	(−1.73)	(0.77)	(−1.27)	(−4.02)	(9.75)	(−0.27)	(−1.09)	(0.53)
children gender^*^time	−0.290	0.358	−0.130	−0.103	0.039	−0.219^**^	0.012	0.081	−0.029
	(−1.36)	(0.48)	(−0.56)	(−1.03)	(0.13)	(−2.65)	(0.45)	(0.98)	(−0.92)
childbirth^*^time	0.092^*^	−0.520^***^	−0.025	0.040	0.137	0.048	0.030^***^	0.087^***^	−0.015
	(2.13)	(−3.56)	(−0.63)	(1.37)	(1.60)	(1.97)	(4.40)	(3.62)	(−1.72)
dadbirth^*^time	0.034	0.153	0.016	0.008	0.057	−0.023	−0.003	0.024	0.009^*^
	(1.78)	(1.73)	(0.85)	(0.33)	(0.84)	(−1.10)	(−0.76)	(1.68)	(2.09)
dad education year^*^time	0.005	−0.159^*^	−0.017	−0.005	−0.002	0.026^**^	−0.002	−0.017	−0.001
	(0.31)	(−2.36)	(−0.76)	(−0.40)	(−0.03)	(3.18)	(−0.47)	(−1.44)	(−0.14)
mombirth^*^time	0.006	−0.004	−0.018	−0.004	−0.077	0.027	0.003	−0.018	−0.003
	(0.32)	(−0.07)	(−0.92)	(−0.20)	(−1.33)	(1.62)	(0.72)	(−1.05)	(−0.47)
mom education year^*^time	−0.025	0.031	−0.025	−0.013	0.056	−0.037^***^	−0.003	0.003	0.009
	(−1.89)	(0.41)	(−1.02)	(−1.25)	(1.29)	(−4.60)	(−0.80)	(0.30)	(1.95)
mountainous terrain^*^time	−0.143	1.391	0.614^*^	0.216^*^	0.495	0.264^*^	0.006	−0.093	−0.040
	(−0.54)	(1.37)	(2.50)	(1.99)	(0.87)	(2.55)	(0.17)	(−0.86)	(−0.89)
_cons	−41.560^**^	124.600^*^	8.700	−16.980	−39.780	−20.110^*^	0.139^***^	6.539^***^	0.530^***^
	(−2.86)	(2.26)	(0.75)	(−1.57)	(−1.27)	(−2.45)	(4.79)	(53.34)	(12.35)
Obs	502	502	502	958	958	958	3,728	3,728	3,728
Adj R-square	0.644	0.652	0.643	0.463	0.271	0.409	0.277	0.130	0.089

* *p* < 0.1,

** *p* < 0.05,

*** *p* < 0.001.

The results show that agricultural disasters have a significant impact only when children are in a non-key class. The coefficient of agricultural disasters occurring is −2.153 and significantly negative at the 0.1% level when it comes to rural children's noncognitive skill, which indicates that the occurrence of agricultural disasters significantly reduces the noncognitive skill of non-key class children, in terms of economic significance; if the municipality that children live in suffered agricultural disasters in the last 2 years, the scores of their noncognitive skills would decrease by 2.153 points. Regarding non-key class children's academic pressure, the coefficient of agricultural disasters occurring is 0.782 and significantly negative at the 0.1% level, which indicates that if the municipality that children live in suffered agricultural disasters in the last 2 years, their academic pressure would increase by 0.782 points. The other independent variables and other class types were not significant.

#### Parents' concern for education

As children's development may be influenced by the family environment, this study analyzed the heterogeneity of children based on different parental concerns. According to the difference in parents' concerns, the children were divided into two groups: in one group, their parents often checked their homework (parents think highly of education), and in the other, their parents ignored their homework (parents think little of education). The results of class-type heterogeneity analyses are presented in Table 4.

**Table 4:** The results of different parents concerns heterogeneity analysis.

**Family education types**	**Parents think little of study**			**Parents think highly of study**		
**Variable**	**Cognitive skill**	**Noncognitive skill**	**Academic pressure**	**Cognitive skill**	**Noncognitive skill**	**Academic pressure**
agriculture disasters occurring	−0.021	−0.817^**^	0.157	0.387	1.983	1.184^***^
	(−0.41)	(−3.13)	(0.71)	(0.99)	(1.86)	(5.35)
_cons	−0.0718	6.716^***^	0.520^***^	0.378^***^	6.051^***^	0.367^***^
	(−1.31)	(29.44)	(8.31)	(3.89)	(20.48)	(4.58)
Obs	3,181	3,181	3,181	2,007	2,007	2,007
Adj R-square	0.357	0.106	0.101	0.261	0.153	0.143

*p < 0.1,

** *p* < 0.05,

*** *p* < 0.001.

The results of the class type heterogeneity analysis show that only when parents think highly of study will agricultural disasters have a significant impact on their noncognitive skills. The coefficient of agricultural disasters occurring is −0.817 and significantly positive at the 5% level when it comes to rural children's noncognitive skill, which indicates that the occurrence of agricultural disasters significantly reduces the noncognitive skill when parents think highly of study, in terms of economic significance. In the municipalities in which children who parents think highly of study live in suffered agriculture disasters in the last 2 years, their measured noncognitive skills might decrease by 0.817 points.

For children whose parents think little of study, the coefficient of agricultural disasters occurring is 1.184 and is significantly negative at the 5% level, which indicates that if the municipality whose parents think little of study has suffered agriculture disasters in the last 2 years, their academic pressure would increase by 1.184 points. Other independent variables were not statistically significant.

## Discussion and conclusions

Using survey data from the CFPS for 2010–2018 and a fixed affected panel model, this study evaluated the effects of agricultural disasters on rural children's development. To verify the results of the full sample, we took a sub-sample of this panel data with three or more occurrences to test the results for cognitive and noncognitive skills. Robustness tests corroborated our full-sample regression results. To further explore the impact of agricultural disasters on children in different school and family environments, we conducted two heterogeneity analyses. The findings are as follows.

This study explored the effects of agricultural disasters on rural children's cognitive and noncognitive skills, and academic pressure. **First**, agricultural disasters negatively impact rural children's cognitive skills, noncognitive skills, and academic pressure. Regarding children's cognitive skills, investment theory points out that to maximize their own utility, parents will selectively invest material and time in their children's human capital (65). Therefore, when agricultural disasters occur, rural parents may have to put more material and time into the resumption of household living operations, so that children will have less material and time investment from the family to develop their cognitive skills (38). As for children's noncognitive skills, this result validates the family stress theory (66), which states that when agricultural disasters occur, rural families face economic hardships that affect their parenting ability and psychological well-being. Under a low level of parental psychological well-being, children's noncognitive skills are influenced because parents are less able to promote family functioning and adopt a parenting style (67). As for children's academic pressure, existing studies have shown that their behavior can be affected by household income because a decrease in household income will affect their emotional and behavioral health and personality trait development (68). When children have problems with their behavioral health and personality trait development, they may not concentrate properly in class or relate well with their classmates (69). This makes the school environment unconducive to children's academic progress and self-recognition, which leads to an increase in children's academic stress (70). In addition, the family stress theory also makes sense here (66); during agricultural disasters, parental pressure will increase and the level of family parenting ability will decrease, so children's stress will increase (71). When children spent a certain amount of time in desperate situations, they felt more depressed during their studies.

**Second**, the effect of agricultural disasters on rural children's development differs according to their family environment. Only when parents thought highly of education did agricultural disasters have a significant and negative impact on children's noncognitive skills. However, for children whose parents thought little of education, agricultural disasters had a significant and positive impact on their academic pressure. We speculate that parents who thought little of their children's studies came from low-income families; they were busy earning money to support their families and did not have extra time to pay attention to their children's homework (72). When agricultural disasters occur, losses in agriculture may put more financial pressure on families, and family stress and parental emotions can be passed on to children, which decreases their noncognitive skills (73); that is, it decreases their noncognitive skill score. Meanwhile, parents who highly thought of their children's studies may have come from higher-income rural families, and when it came to agricultural disasters, they were not hit too hard. Parents still had enough energy to pay attention to their children's studies, which led to an increase in their children's academic pressure.

**Third**, the effect of agricultural disasters on rural children's development differs according to their school environment. In the key and ordinary class groups, agricultural disasters do not have any significant impact on rural children's outcomes; in the non-key class group, agricultural disasters have a significant and positive impact on children's noncognitive score and academic pressure, that is, when the district children live in has experienced agricultural disasters in the last 2 years, their noncognitive skill level will decrease and academic pressure will increase. We speculate that the reason for this is that the children in the no-key class had a worse learning base than those in the other classes, so they may have faced more academic pressure. Unsatisfactory academic performance and high levels of academic stress can affect children's mental health; thus, children's noncognitive skills are also affected (74).

Based on these results, our study offers several implications for enhancing the development of rural children suffering the impact of agricultural disasters. **First**, the significant role of agricultural disasters in decreasing the level of rural children's development should be emphasized. We found that agricultural disasters had a negative impact on rural children's cognitive and noncognitive skills and academic pressure. Therefore, we should strengthen disaster prevention and mitigation, take measures in agricultural natural disaster planning and early warning, and minimize the losses to rural families caused by agricultural disasters, thus reducing the impact on children's development. As such, faced with the impact of agricultural disasters, which leads to insufficient development resources that families can provide to children, the government should make more efforts to enhance rural children's protection and improve their developmental conditions in rural China.

**Second**, the negative impact of agricultural disasters on children's development also confirms that fluctuations in agricultural income lead to a decline in farming households' investment in children's human capital, which diminishes children's well-being and human capital (15). As investments in children are the cornerstone of enhancing well-being and breaking the cycle of intergenerational transmission of poverty, and are also central to national growth and economic development, we strongly encourage policymakers and donors to fund research on the dissemination of new crop varieties that are more resilient to climate volatility with high-yield traits to better ensure farmers' income, which will enable them to continue spending money on and paying attention to their children's development.

**Third**, children's environment also plays an important role in their development, which is shown by heterogeneity. Both the school and family environments may influence children's development. Policymakers should focus on improving the schools and home environments of rural children. The Ministry of National Education should strengthen the construction of schools and investment in educational resources in rural areas, rationally allocate class-teaching resources, and narrow the academic gap between students. The importance of family education in rural areas should be emphasized, and publicity and education should be enhanced to raise parents' awareness of the importance of a supportive family atmosphere for children's development.

**Fourth**, since the resources and support needed for rural children's development are closely associated with farm household income, which is strongly influenced by agricultural disasters, to minimize farming household losses brought about by agricultural disasters, related institutions, such as the Ministry of Agriculture and Rural Affairs of the People's Republic of China, China Meteorological Administration, and Ministry of Human Resources and Social Security of the People's Republic of China, can establish linkage mechanisms for agricultural disasters and emergency management systems for natural disasters to reduce the impact of damage and losses on farmers' income and safeguard their livelihoods before, during, and after stages.

**Last**, the findings highlighted the extended period of the impact of agricultural disasters and identified important opportunities for intervention in human capital development resources to enable children to fulfill their potential, while previous findings in the literature on the lack of impact of agricultural disasters on children's development should be reviewed. Without targeted interventions, the children's future development may be compromised. The results should draw the attention of child development-related institutions, such as the Ministry of Education of the People's Republic of China and the National Working Committee on Children and Women under the State Council, to children's human capital development and security benefits. Furthermore, this study provides guidance for future research to discover and validate the impact mechanisms of agricultural disasters on rural children's development.

Limited by the available data, our study did not have perfectly balanced panel data, and the identification of agricultural disasters was limited to the regional level rather than the individual level; that is, where a specific household (or child) actually experienced agricultural disasters. However, since we controlled for individual fixed effects as much as possible, our results provide new insights and innovative findings on the effects of agricultural disasters on rural children's development. In subsequent studies, we will continue to search for better data to explain in greater depth the effects of agricultural disasters on rural children's development. This series of studies will support the understanding of the relationships between agricultural disasters and child development and the long-term impacts of agricultural disasters on the human life cycle. At the practical application level, this series of studies can help children who experience agricultural disasters deal with likely negative consequences.

## Paper context

In rural China, frequent agricultural disasters negatively impact family production and life, posing a barrier to children's development. This pattern increases the difficulty of narrowing the urban-rural development gap. Our study evaluated the effects of agricultural disasters on rural children's development, including their cognitive skills, noncognitive skills, and academic pressure. Special attention should be paid to the consequences of disasters on children, and appropriate measures should be taken to reduce possible negative impacts.

## Data availability statement

Publicly available datasets were analyzed in this study. This data can be found at: the Institute of Social Science Survey (ISSS) at Peking University (http://www.isss.pku.edu.cn/cfps/index.htm).

## Author contributions

SX analyzed the data and wrote first draft of the manuscript. PZ provided overall supervision of the project, reviewed the final version of this manuscript, and agreed to its submission for publication. All authors conceptualized the study, provided training and technical support during data collection, and provided intellectual input to the manuscript development.

## Funding

The research is funded by the National Social Science Foundation of China (CN) [Grant Number: 19ZDA116], the Fundamental Research Funds for the Central Universities of China, and Center of Social Welfare and Governance in Zhejiang University.

## Conflict of interest

The authors declare that the research was conducted in the absence of any commercial or financial relationships that could be construed as a potential conflict of interest.

## Publisher's note

All claims expressed in this article are solely those of the authors and do not necessarily represent those of their affiliated organizations, or those of the publisher, the editors and the reviewers. Any product that may be evaluated in this article, or claim that may be made by its manufacturer, is not guaranteed or endorsed by the publisher.
